# Audit on the Appropriateness of Monitoring Frequencies and Clinical Escalation From National Early Warning Scores 2 (NEWS2) in Old Age Psychiatry Patients

**DOI:** 10.1192/bjo.2025.10610

**Published:** 2025-06-20

**Authors:** Vanessa Tolulope Jacobs, Wan Yee Ko, Monira Miah, Ivan Shanley

**Affiliations:** Essex Partnership University NHS Foundation Trust, Rochford, United Kingdom

## Abstract

**Aims:** Physical health monitoring and assessment in psychiatry is fundamental to holistic care provision, and NEWS2 provide an objective, standardised pathway that aids the timely detection of clinical deterioration. This subsequently facilitates appropriate clinical review and care escalation.

This audit based on an older adult functional psychiatric ward aimed to evaluate the adherence to NEWS2 protocols in the following domains:

Monitoring frequency.

Clinical responses to NEWS2.

**Methods:** A thorough review of NEWS2 documentation for 24 patients on Beech Ward at Rochford Hospital over the same time period of 3 weeks was completed. Of these patients, 12 were present for this same time period, and 3 of these patients were omitted due to frequent refusal of clinical observations. This review yielded 476 NEWS2 entries from 9 patients between ages 68–86, with a gender ratio of 4:5 male to female and admission durations from 44–355 days. The aggregate score for each NEWS2 entry was collated, and the appropriateness of monitoring frequency was assessed by directly comparing the documented monitoring frequency to the Royal College of Physicians’ recommended monitoring frequency. To evaluate the appropriateness of clinical responses to the aggregate score, care escalation documentation for each NEWS2 entry alongside corresponding clinical documentation of patient reviews by nursing staff and doctors were assessed.

**Results:** This study yielded 476 NEWS2 entries – 42% demonstrated appropriate monitoring frequencies, with most adherence to 12-hourly routine monitoring due to NEWS 0 (81%). The remaining 58% of entries evidenced monitoring frequencies which deviate from standard recommendations, with all of these observations monitored at frequencies less than the recommended minimum. Recommended minimum 1-hourly observations were monitored up to 12-hourly, minimum 4-hourly observations were monitored up to 30-hourly, and minimum 12-hourly observations were monitored up to 48-hourly. Inappropriate clinical responses to patient escalation were secondary to incomplete documentation of care escalation, and lack of escalation to the medical team for clinical review in light of a score of 3 in a single parameter.

**Conclusion:** In conclusion, these findings highlight the need for better adherence to recommended monitoring frequencies to promote patient safety and care, as evidenced by the deviation in monitoring intervals. Clinical nursing responses to NEWS2 were appropriate, however, completeness of documentation is imperative to ensure care escalation is not overlooked. This has prompted discussions with the multidisciplinary team regarding adherence to NEWS2 documentation recommendations, and intradepartmental teaching sessions outlining clinical handover and indications for care escalation.

